# *Dictyostelium *cells bind a secreted autocrine factor that represses cell proliferation

**DOI:** 10.1186/1471-2091-10-4

**Published:** 2009-02-02

**Authors:** Jonathan M Choe, Deenadayalan Bakthavatsalam, Jonathan E Phillips, Richard H Gomer

**Affiliations:** 1 Department of Biochemistry and Cell Biology, MS-140, Rice University, Houston, TX 77005-1892, USA

## Abstract

**Background:**

*Dictyostelium *cells secrete the proteins AprA and CfaD. Cells lacking either AprA or CfaD proliferate faster than wild type, while AprA or CfaD overexpressor cells proliferate slowly, indicating that AprA and CfaD are autocrine factors that repress proliferation. CfaD interacts with AprA and requires the presence of AprA to slow proliferation. To determine if CfaD is necessary for the ability of AprA to slow proliferation, whether AprA binds to cells, and if so whether the binding requires the presence of CfaD, we examined the binding and effect on proliferation of recombinant AprA.

**Results:**

We find that the extracellular accumulation of AprA increases with cell density and reaches a concentration of 0.3 μg/ml near a stationary cell density. When added to wild-type or *aprA*^- ^cells, recombinant AprA (rAprA) significantly slows proliferation at 0.1 μg/ml and higher concentrations. From 4 to 64 μg/ml, the effect of rAprA is at a plateau, slowing but not stopping proliferation. The proliferation-inhibiting activity of rAprA is roughly the same as that of native AprA in conditioned growth medium. Proliferating *aprA*^- ^cells show saturable binding of rAprA to 92,000 ± 11,000 cell-surface receptors with a *K*_*D *_of 0.03 ± 0.02 μg/ml. There appears to be one class of binding site, and no apparent cooperativity. Native AprA inhibits the binding of rAprA to *aprA*^- ^cells with a *K*_*i *_of 0.03 μg/ml, suggesting that the binding kinetics of rAprA are similar to those of native AprA. The proliferation of cells lacking CrlA, a cAMP receptor-like protein, or cells lacking CfaD are not affected by rAprA. Surprisingly, both cell types still bind rAprA.

**Conclusion:**

Together, the data suggest that AprA functions as an autocrine proliferation-inhibiting factor by binding to cell surface receptors. Although AprA requires CfaD for activity, it does not require CfaD to bind to cells, suggesting the possibility that cells have an AprA receptor and a CfaD receptor, and activation of both receptors is required to slow proliferation. We previously found that *crlA*^- ^cells are sensitive to CfaD. Combined with the results presented here, this suggests that CrlA is not the AprA or CfaD receptor, and may be the receptor for an unknown third factor that is required for AprA and CfaD activity.

## Background

Much remains to be understood about the mechanisms that regulate the size of a tissue. In some cases, it appears that secreted diffusible factors allow cells in a group to sense the size of the group [1,2]. As the number of cells secreting the factor increases, the concentration of the factor increases [1,3]. The cells sense the concentration of the factor, allowing them to sense the size of the group of cells. If the factor inhibits cell proliferation, the resulting negative feedback loop could effectively stop proliferation once a specific group or tissue size is reached. The group or tissue size would then be determined by how much factor the cells secrete, the diffusion properties of the factor, and how sensitive the cells are to the factor. There are a few examples of this sort of negative feedback loop. For example, myostatin is a protein secreted by muscle cells, and myostatin concentrations rise as the amount of muscle in the body increases [4]. Myostatin inhibits myoblast proliferation, which keeps the amount of muscle in the body at a relatively constant level [5]. Mutation or disruption of myostatin results in abnormally large muscles [6,7]. Another example of a negative feedback loop is thyroid size regulation. Thyroid cells secrete thyroid hormone, which inhibits the release of thyroid-stimulating hormone [8] from the pituitary. TSH functions to stimulate the growth of the thyroid. Thus, if the thyroid is damaged, thyroid hormone levels would fall, allowing more TSH release to promote thyroid growth [9]. A third example of a negative feedback loop involves regulation of adipose tissue within the human body. The leptin protein is secreted by adipocytes and signals the amount of adipose tissue present in the body [10,11]. High leptin levels signal to the body that appetite is satisfied, which decreases adipose tissue accumulation to complete the feedback loop.

There are many tissues where there is evidence for the existence of a secreted factor that inhibits cell proliferation to regulate tissue size, but the identity of the factor and its signal transduction pathway is unknown. For example, in mammals the liver has the ability to regenerate to the correct size if any portion of the liver is removed, and this appears to be mediated by an unknown factor that is secreted into the blood [12]. The spleen is another example of a tissue whose size appears to be negatively regulated by unknown secreted factors [13]. Identifying these factors and their signal transduction pathways will aid in our understanding of tissue size regulation.

*Dictyostelium discoideum *is an excellent model system to study secreted factors and the regulation of proliferation and group size. *Dictyostelium *is a haploid unicellular eukaryote that feeds on soil bacteria. There are several secreted signals whose extracellular concentration is sensed by *Dictyostelium *cells to, in turn, sense the local density or number of other *Dictyostelium *cells. When cells starve, they stop dividing and begin secreting an 80 kDa glycoprotein called conditioned medium factor (CMF) [3,14-18]. As more and more cells in a population starve, the extracellular CMF concentration rises. When there is a high percentage of starved cells, as indicated to the cells by a high extracellular concentration of CMF, the cells aggregate to form multicellular structures called fruiting bodies. The aggregating cells form dendritic streams flowing toward a common center. To regulate the size of the fruiting bodies, the streams break up into groups if there are too many cells in a stream [19]. Cells sense if there are too many cells in a stream by sensing the concentration of counting factor (CF), a protein complex secreted by the aggregating cells [20-25].

CF is a 450 kDa complex of at least 4 different proteins [20,23,26-28]. Partially purified CF contains 8 proteins, and we have been systematically identifying which are true CF components and which are contaminants. We identified two proteins, AprA and CfaD, in the partially purified CF preparation that are not CF components [29,30]. AprA and CfaD are components of a 150 kDa complex and appear to bind to each other [30]. Disruption of either *aprA *or *cfaD *results in cells that have an abnormally high proliferation rate, while overexpression of either protein slows proliferation [29,30]. Adding either 10 ng/ml immunoprecipitated native AprA (at the time, we had not found conditions to make recombinant AprA) [29], or 20 ng/ml or higher concentrations of recombinant CfaD [30], also slows cell proliferation. Recombinant CfaD however does not affect the proliferation of *aprA*^- ^cells, suggesting that CfaD needs the presence of AprA to inhibit proliferation [30]. Neither AprA nor CfaD affect growth rates per nucleus (effectively the mass increase per hour of cells) [29,30]. Because of the finite amount of available nutrients in a given patch of soil, and because cells will soon starve after they reach a high cell density, we have hypothesized that the functions of AprA and CfaD are to slow proliferation without slowing growth as the cells reach high density, so that when the cells do starve, the cells will tend to be large and have a relatively large store of nutrients [29,30].

While studying novel proteins with similarities to G-protein-coupled receptors, Raisley et al. [31] found that cells lacking CrlA, a putative a G protein coupled receptor, proliferate faster than wild type cells. Interestingly, we found that compared to its effect on wild-type cells, recombinant CfaD weakly inhibits the proliferation of *crlA*^- ^cells [30]. This suggested that CrlA potentiates, but is not necessary for, CfaD signal transduction.

We recently found conditions in which we can express recombinant AprA (rAprA) [30]. In this report, we show that rAprA slows the proliferation of wild-type and *aprA*^- ^cells, but has no effect on *cfaD*^- ^or *crlA*^- ^cells. However, rAprA binds to all four cell types, suggesting that CfaD and CrlA are necessary for AprA signal transduction, and that CrlA is a receptor for a different factor that regulates the ability of AprA to act as a chalone.

## Methods

### Cell culture

Wild-type Ax2, *aprA*^- ^strain DB60T3-8 [29], *cfaD*^- ^strain DB27C-1 [30], and *crlA*^- ^strain JH557 [31] were cultured following Brock et al. [20] in HL5 medium (Formedium Ltd., Norwich, England). The growth of NC4 on bacteria was done as described in [30]. Calculation of doubling times was done as previously described [29].

### Recombinant AprA and CfaD Expression and Purification

Recombinant AprA (rAprA) and recombinant CfaD (rCfaD) were prepared following Bakthavatsalam et al. [30]. The concentrations of the purified proteins were determined as described in Gao et. al., [32].

### Quantification of secreted AprA

The conditioned growth medium samples used for the AprA quantitation were aliquots of the samples we previously used to measure the accumulation of CfaD [30], allowing a direct comparison of the amount of AprA and the amount of CfaD secreted by cells. Samples of the conditioned growth media were run on 4–15% acrylamide gels (Biorad Laboratories, Hercules, CA) along with different known concentrations of rAprA. Western blots were stained with affinity-purified anti-AprA antibodies as described previously [29]. The AprA bands were then scanned and analyzed using ImageJ . The concentration of secreted AprA at each cell density was quantified by comparing against the known concentrations of rAprA.

### Proliferation inhibition by rAprA or conditioned growth medium

To test the biological activity (cell proliferation inhibition activity) of rAprA or conditioned growth medium (prepared from wild-type cells grown to 1.2 × 10^7 ^cells/ml, where the measured rAprA concentration in the conditioned growth medium is 0.3 μg/ml), cells were grown in HL5 media to a density of 2 × 10^6 ^cells/ml, collected by centrifugation at 1,500 × g for 3 minutes, and resuspended in HL5 media to 5 × 10^5 ^cells/ml. Cells were then counted before and after 12 hours of incubation with rAprA (or an equal volume of buffer as a control) or conditioned growth medium (or an equal volume of HL5 as a control). A sigmoidal dose-response curve

$$\text{Percent proliferation}=100-\left(\frac{\text{Max}}{1+10^{\left((\log(\text{EC50}))-(\log(\text{rAprA concentration))}\right)}}\right)$$

was then fit to the data to obtain Max and EC50 using nonlinear regression with Prism (GraphPad software, San Diego, CA). In Table 1, Max is called 'proliferation as percent of control at high rAprA'. The units/ml of proliferation-inhibiting activity was defined as the fold dilution of added rAprA or conditioned growth medium that caused a 20% decrease in the density of cells after the 12-hour incubation. The units/ml was calculated using the Max and EC50 values obtained from the above curve fitting, and solving for rAprA concentration with percent proliferation set to 80. For a known concentration of AprA, the units/ml of activity could then be converted to units/μg.

**Table 1:** rAprA and wild-type conditioned growth medium slow the proliferation of wild-type (WT) and *aprA*^- ^cells.

	**rAprA**		**WT conditioned growth medium (CGM)**		
	
**Cell type**	**Activity, units/μg**	**Proliferation as percent of control at high rAprA**	**Activity, units/ml**	**Proliferation as percent of control at high CGM**	**Activity, units/μg AprA**
**WT**	3.7 ± 0.9	71 ± 1	1.5 ± 0.5	64 ± 8	4.9 ± 1.7
***aprA*^-^**	10.7 ± 2.8	70 ± 1	1.4 ± 0.5	60 ± 6	4.5 ± 1.7
***cfaD*^-^**	0 ± 0	97 ± 1	ND	ND	ND
***crlA*^-^**	0 ± 0	97 ± 1	ND	ND	ND

Cells of the indicated strain were grown in the presence of different concentrations of rAprA, and cells were counted after 12 hours. After fitting sigmoidal dose response curves (Figure 2), the activity of rAprA and the proliferation as a percent of control at high concentrations of rAprA were calculated. Similar fits were done for cells grown in different dilutions of wild-type conditioned growth medium (Figure 3). The activity of the AprA in conditioned growth medium was calculated using the observed AprA concentration of 0.3 μg/ml. All values are mean ± SEM from 3 independent experiments. ND indicates not determined.

### Determination of optimal binding time for rAprA

To determine the saturation binding time of rAprA, cells were grown to a density of 2 × 10^6 ^cells/ml. Cells were collected by centrifugation at 1,500 × g for 3 minutes. Cells were briefly washed twice in ice cold HL5 and were resuspended in ice cold HL5 to a final concentration of 1.0 × 10^7 ^cells/ml and kept on ice. 0.5 μl of 300 μg/ml rAprA was added to 500 μl of cells, and this was gently mixed on a rotator at 4°C for 0, 1, 2, 5, 10, or 30 minutes. Cells were collected after the indicated times by centrifugation at 10,000 × g for 30 seconds and washed briefly in 500 μl of ice cold HL5. Following the wash, the cells were resuspended in 100 μl of SDS sample buffer and heated at 95°C before loading 10 μl onto a 4–15% gel (Biorad). Different concentrations of rAprA were used as a standard on the same gel, and proteins were transferred onto a PVDF membrane (Immobilin-P, Millipore corporation, Bedford, MA). A duplicate gel was stained with Coomassie to verify that there were roughly equal amounts of protein in each sample. To detect rAprA (which contains a myc tag), the blots were stained with a 1:10,000 dilution of anti-myc antibodies (Bethyl laboratories, Montogomery, TX) in 25 mM Tris/HCl pH 7.4, 150 mM NaCl/0.1% Tween-20 for 1 hour, and subsequent steps for Western blotting were done following [29]. The rAprA bands on the autoradiograph were scanned and the binding experiment intensities were compared against the standards to determine the concentration of bound rAprA. An association binding curve

Bound rAprA = Bmax(1-e^-kt^)

where t is time was then fit to the data using Prism.

### Steady state binding

A binding assay was performed as described above except that the cells were incubated with different concentrations of rAprA for 10 minutes at 4°C. One- and two- site binding curves with and without cooperative binding were then fit to the data using Prism.

### Competitive binding

For competitive binding assays, wild-type conditioned growth medium (CGM) was prepared as described previously [33]. The concentration of AprA in the conditioned medium was measured as described above. A binding assay was performed as described above with the exception that *aprA*^- ^cells were used, and were resuspended in pre-chilled mixtures of HL5 medium and wild type conditioned growth medium before adding 150 ng/ml of rAprA. After 10 minutes of incubation, the amount of bound rAprA was determined as described above. A sigmoidal dose-response curve

$$\text{Bound rAprA}=\text{BT}-\left(\frac{\text{BT}}{1+10^{\left((\log(\text{IC50}))-(\log(\text{native AprA concentration))}\right)}}\right)$$

where BT is the maximal rAprA binding in the competition assay and IC50 is the concentration of native AprA that causes 50% inhibition of the AprA binding, was then fit to the data using nonlinear regression with Prism. The K_i _for the binding inhibition was then calculated from the IC50 using the equation of Cheng and Prusoff [34].

## Results

### Recombinant AprA is bioactive

**Figure 1:**
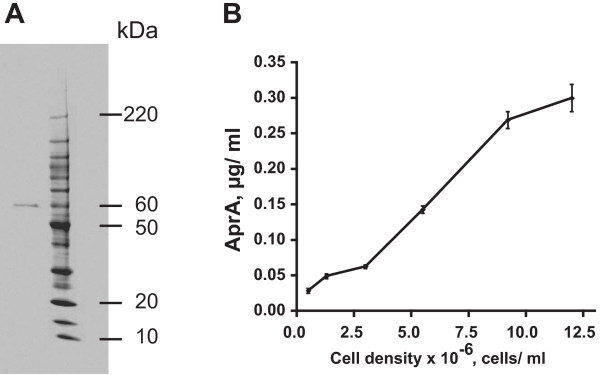
**The concentration of extracellular AprA increases with cell density**. **A) **An SDS-polyacrylamide gel of recombinant AprA (left lane) and molecular mass standards (right lane) was stained with Coommassie.**B) **Using different concentrations or recombinant AprA to make a standard curve, Western blots were used to determine the extracellular concentration of AprA as a function of cell density. Values are mean ± SEM (n = 3). The absence of error bars indicates that the error was smaller than the plot symbol.

AprA is a secreted signal in *Dictyostelium *cells that slows cell proliferation [29]. To determine the extracellular concentration of AprA, we expressed and purified recombinant AprA (rAprA) for use as a reference standard (Figure 1A). The rAprA appeared as a single band at 60 kDa, which roughly corresponds to the sum of the predicted molecular mass of the his/myc tag on the rAprA (5.3 kD) and the predicted mass of the secreted portion of the AprA polypeptide backbone (53.1 kDa). Since the observed mass of the secreted portion of native AprA is 60 kDa [29], the observed mass of rAprA suggests that the secreted native AprA contains ~5–7 kDa of posttranslational modification, presumably glycosylation. Western blots of conditioned growth medium electrophoresed alongside known quantities of rAprA were stained with affinity-purified anti-AprA antibodies. We observed an increase in the accumulation of extracellular AprA with cell density during the growth of wild-type cultures (Figure 1B). As the cultures reached saturation at ~1.2 × 10^7 ^cells/ml, the AprA concentration rose to 0.3 μg/ml. This corresponds to an accumulation of 2.5 × 10^-8 ^μg/cell. Similar assays showed that at a density of 2 × 10^6 ^cells/ml, both *cfaD*^- ^and *crlA*^- ^cells had accumulated 0.6 ± 0.1 μg/ml of extracellular AprA (data not shown), indicating that loss of CfaD or CrlA causes cells to accumulate, compared to wild-type cells, roughly ten times more extracellular AprA.

It took 16 hours for wild-type cultures to proliferate from 0.5 × 10^6 ^cells/ml to 1.3 × 10^6 ^cells/ml, and during this time the extracellular AprA concentration increased by 0.021 ± 0.001 μg/ml (mean ± SEM, n = 3). Assuming that during log-phase growth the cell density ρ = N_o_e^kt^, we see that with N_o _= 0.5 × 10^6 ^cells/ml, k = 0.060/hour. Assuming a constant AprA accumulation rate/cell/hour = X, then

$$21\text{ ng/ml}=\int_{0}^{16}X\rho dt$$

Solving for X, we find that between 0.5 × 10^6 ^and 1.3 × 10^6 ^cells/ml, the extracellular AprA accumulation rate is 1.6 × 10^-9 ^μg/cell/hour, or 2.6 × 10^-11 ^μg/cell/minute, or 260 molecules of AprA/cell/minute. Similar calculations were done for the other cell density ranges shown in Figure 1B, as well as for the extracellular CfaD concentrations shown in Figure 3 of [30]. As shown in Table 2, with the assumption that there is no breakdown of extracellular AprA or CfaD, the accumulation rate of extracellular AprA per cell per hour fluctuates as the cell density in the population increases, with a general tend of decreasing as the cells approach saturation density. Conversely, the accumulation rate of extracellular CfaD per cell per hour increases as the cell density in the population increases (Table 2).

**Table 2:** The accumulation of AprA and CfaD as a function of cell density.

	**AprA accumulation**		**CfaD accumulation**	
	
**Density range, 10^6 ^cells/ml**	**10^-10 ^μg/cell/hour**	**Molecules/minute**	**10^-10 ^μg/cell/hour**	**Molecules/minute**
**0.5 – 1.3**	15.4 ± 0.6	260 ± 10	< 0.004	< 1
**1.3 – 3.0**	2.9 ± 0.1	49 ± 2	0.7 ± 0.1	11 ± 2
**3.0 – 5.5**	8.5 ± 0.7	140 ± 10	1.0 ± 0.2	17 ± 3
**5.5 – 9.2**	7.3 ± 0.4	120 ± 7	1.2 ± 0.1	19 ± 2
**9.2 – 12**	1.3 ± 0.3	22 ± 5	3.6 ± 0.2	59 ± 4

The accumulation values are derived from the AprA quantitation in Figure 1 and from the CfaD quantitation in Figure 3 of [30]; both figures used the same set of samples for quantitation. Values are mean ± SEM from 3 separate experiments.

In the wild, *Dictyostelium *cells grow on soil surfaces. The parental strain used in these studies is an axenic strain derived from an isolate from North Carolina called NC4 [35]. We found that when NC4 strains grow on lawns of bacteria on agar plates, they secrete both CfaD and AprA, and that NC4 cells growing on bacteria accumulate approximately 4 times more CfaD per cell than Ax2 cells at 1.2 × 10^7 ^cells/ml in shaking culture [30]. Using rAprA to generate a standard curve, we found that when there are 3 × 10^7 ^NC4 cells on an agar plate, the agar contains 2.0 ± 0.1 μg of AprA (mean ± SEM, n = 3). This corresponds to an accumulation of 6.6 × 10^-8 ^μg/cell, which is approximately 2.6 times higher than the accumulation for Ax2 cells at stationary phase. The data thus suggest that in the natural environment, cells accumulate somewhat more AprA and CfaD than axenic cells in shaking culture.

**Figure 2:**
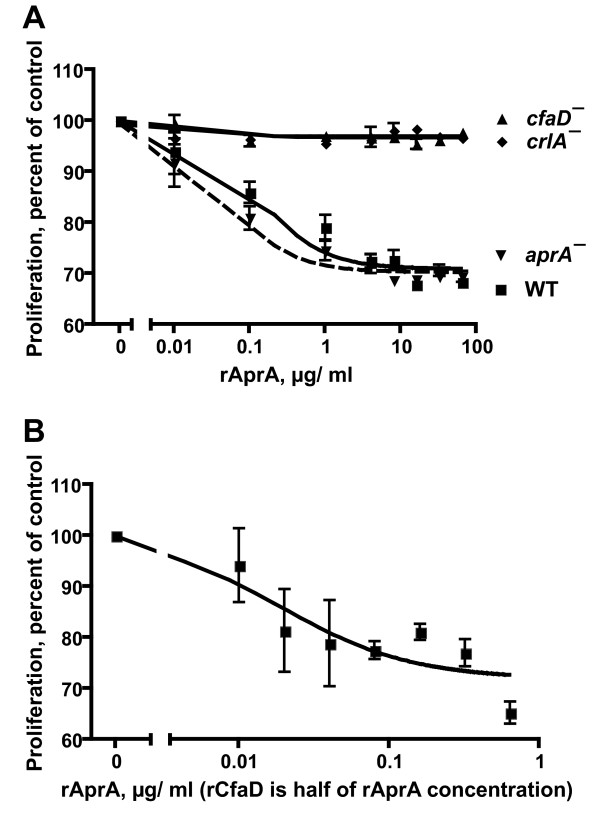
**rAprA slows cell proliferation**. **A) **Different concentrations of rAprA were added to the indicated cell types, and after 12 hours cells were collected and counted. WT is wild-type. Values are mean ± SEM (n = 3). The absence of error bars indicates that the error was smaller than the plot symbol. The lines are sigmoidal dose response curve fits to the data; the dashed line is the fit to *aprA*^-^. The inhibition of wild type and *aprA*^- ^proliferation is significant with p < 0.01 at 0.1 μg/ml and higher rAprA concentrations (1-way ANOVA, Dunnett's test). **B) **A 2:1 mixture of rAprA and rCfaD was added to wild-type cells as in **A **above, so that at, for instance, 0.32 μg/ml rAprA there was an additional 0.16 μg/ml rCfaD. Values are mean ± SEM (n = 3). The line is a sigmoidal dose response curve fit to the data. The inhibition of wild type proliferation is significant with p < 0.05 at 0.02 μg/ml rAprA/0.01 μg/ml rCfaD and higher concentrations (t test).

We previously observed that immunoprecipated native AprA slows the proliferation of wild-type and *aprA*^- ^cells [29]. To determine if any eukaryote-specific posttranslational modification such as glycosylation is part of the AprA active site, we added rAprA to cells. After 12 hours, rAprA at concentrations at and above 0.1 μg/ml significantly slowed the proliferation of wild-type and *aprA*^- ^cells (Figure 2A and Table 1). A recombinant version of the human serum protein Serum Amyloid P, made with the same expression vector in the same bacterial cell line, as well as bovine serum albumin, had no effect on cell proliferation (data not shown). Even at high concentrations (64 μg/ml), rAprA was only able to slow the proliferation of wild-type and *aprA*^- ^cells rather than completely arrest their proliferation (Figure 2A). The doubling times we observed for wild-type and *aprA*^- ^cells were 12.7 and 9.1 hours respectively, essentially identical to what we previously observed [29]. At 1 μg/ml rAprA, the doubling time for wild-type cells was 19.3 hours, and at 4 μg/ml the doubling time was 24.5 hours, similar to the 23.3 hour doubling time we observed for cells overexpressing AprA [29]. Interestingly, rAprA had essentially no ability to slow the proliferation of *crlA*^- ^or *cfaD*^- ^cells. These results demonstrate that rAprA is bioactive, that if AprA is glycosylated, the glycosylation is not essential for bioactivity, and suggest that CrlA and CfaD are required for the ability of rAprA to slow cell proliferation.

### CfaD and AprA potentiate each other's ability to slow proliferation

Like AprA, CfaD is a protein secreted by growing *Dictyostelium *cells that slows cell proliferation [30]. CfaD appears to bind to AprA and requires AprA for bioactivity. To determine if rCfaD potentiates the activity of rAprA, we added mixtures of rAprA and rCfaD to cells. CfaD accumulates to ~0.08 μg/ml when cells are at 1.2 × 10^7 ^cells/ml, while AprA accumulates to ~0.3 μg/ml at this density. As a rough comparison, we thus added 2:1 w/w mixtures of rAprA:rCfaD to wild type cells (Figure 2B). A fit of a sigmoidal dose response curve indicated that at high concentrations, the mixture is able to slow proliferation to 72 ± 3% of control (mean ± SEM, n = 3). This is not significantly different from the amount that high concentrations of rAprA or rCfaD can slow proliferation (Table 1 and [30]). rAprA slows proliferation to 80% of control at ~0.27 μg/ml (Figure 2A and Table 1), while rCfaD slows proliferation to 80% of control at 0.05 μg/ml [30]. The mixture slows proliferation to 80% of control at 0.045 μg/ml rAprA/0.022 μg/ml rCfaD (Figure 2B). This suggests that the presence of CfaD decreases the concentration of AprA needed to slow proliferation, and vice versa.

**Figure 3:**
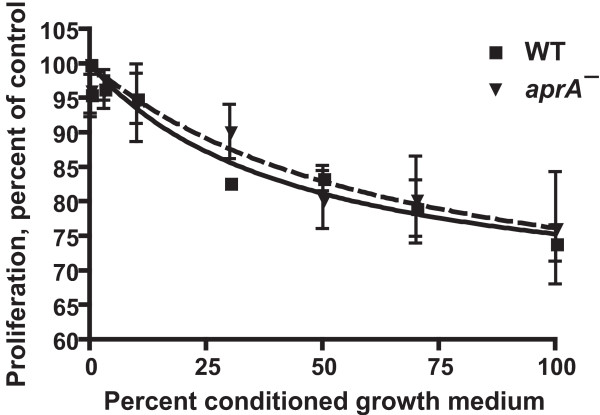
**Wild-type conditioned growth medium slows cell proliferation**. Conditioned growth medium was collected and diluted with fresh growth medium to the indicated concentrations. Wild-type (WT) and *aprA*^- ^cells were then grown in the mixed media, and cells were counted after 12 hours. Values are mean ± SEM (n = 3). The absence of error bars indicates that the error was smaller than the plot symbol. Lines are curve fits of a sigmoidal dose response curve; the dashed line is the fit to *aprA*^-^. The inhibition of WT and *aprA*^- ^proliferation is significant with p < 0.05 at 30% and higher conditioned growth medium concentrations (1-way ANOVA, Dunnett's test).

The conditioned growth medium from wild-type cells slows *aprA*^- ^cell proliferation, whereas conditioned growth medium from *aprA*^- ^cells lacks this activity, indicating that AprA is a key component of the proliferation-inhibiting activity in wild-type conditioned growth medium [29]. To compare the proliferation-inhibiting activity of recombinant AprA to the AprA-associated activity in conditioned growth medium, cells were grown in different dilutions of conditioned growth medium collected from wild-type cells at 1.2 × 10^7 ^cells/ml. After 12 hours, wild-type conditioned growth medium at concentrations above 30% significantly slowed the proliferation of wild type and *aprA*^- ^cells (Figure 3 and Table 1). Using the observed AprA concentration in conditioned growth medium (collected from wild-type cells at 1.2 × 10^7 ^cells/ml) of 0.3 μg/ml (Figure 1), the AprA activity, as measured in units/μg of the AprA in wild-type conditioned growth medium, on wild-type or *aprA*^- ^cells was roughly similar to the activity of rAprA on wild-type cells; the differences were not statistically significant (p > 0.05, 1-way ANOVA, Tukey's test) (Table 1). However, at high concentrations, the wild-type conditioned growth medium caused a somewhat greater inhibition of proliferation than high concentrations of rAprA (Figures 2 and 3 and Table 1). Together, the results suggest that rAprA has roughly the same bioactivity as native AprA, but that there may be additional factors in conditioned growth medium that slow cell proliferation.

### Recombinant AprA binds to *Dictyostelium *cells

**Figure 4:**
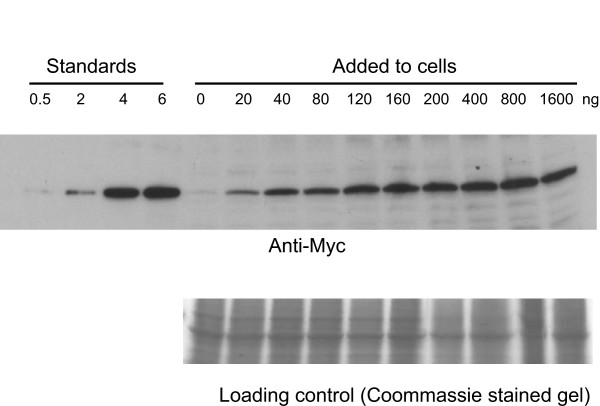
**Binding of rAprA to cells**. For a rAprA binding assay, wild-type cells were incubated for 10 minutes with the indicated concentrations ("Added to cells" in ng/ml) of myc-tagged rAprA. The cells were then washed to remove unbound rAprA, and were solubilized in SDS sample buffer. A western blot of the solubilized cells electrophoresed alongside different amounts of myc-tagged rAprA ("Standards") was stained with anti-myc antibodies (upper panel) while a duplicate gel of the cell samples was stained with Coomassie (lower panel). The heavy band in the Coomassie-stained samples is actin.

**Figure 5:**
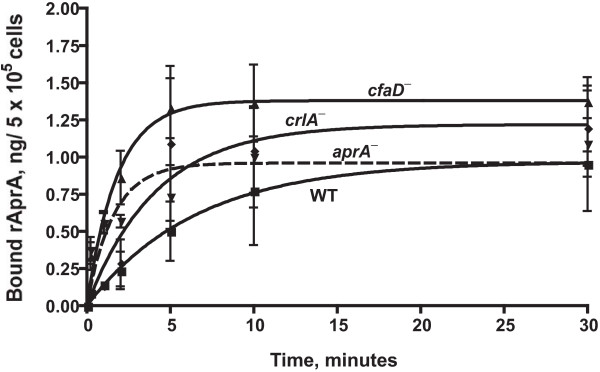
**Time course of rAprA binding to cells**. Cells of the indicated strains (WT is wild-type) were incubated with 150 ng/ml of rAprA for the indicated times at 4°C. Cells were collected and the bound myc-tagged rAprA was quantitated by western blots (staining for the myc tag), using known amounts of rAprA as standards. The plot symbols are the same as those in Figure 2. Values are mean ± SEM (n = 3). Lines are curve fits of an association binding curve; the dashed line is the fit to *aprA*^-^.

To determine if AprA is sensed by cell surface receptors, we examined the binding of rAprA to cells. The binding assays were done in HL5 growth medium, as we previously observed that we could measure binding of CMF to cells in this medium [16]. After trying a variety of binding times and concentrations to establish rough time and concentration conditions for the assays (Figure 4 and data not shown), the time course of rAprA binding was examined to establish steady state conditions for further binding assays. The amount of rAprA bound to cells reached near steady state levels by 10 minutes (Figure 5). Interestingly, even though rAprA was unable to inhibit the proliferation of *cfaD*^- ^and *crlA*^- ^cells, rAprA bound to these cells. Although there appeared to be differences in the binding rates, the difference in the binding time constant k between all pairs of cell types was not significant (p > 0.05, 1-way ANOVA, Tukey's test). In addition, although it appeared that there were differences in the amount of rAprA bound at 10 and 30 minutes to the different cell types, the difference between all pairs of cell types was not significant (p > 0.05, 1-way ANOVA, Tukey's test).

**Figure 6:**
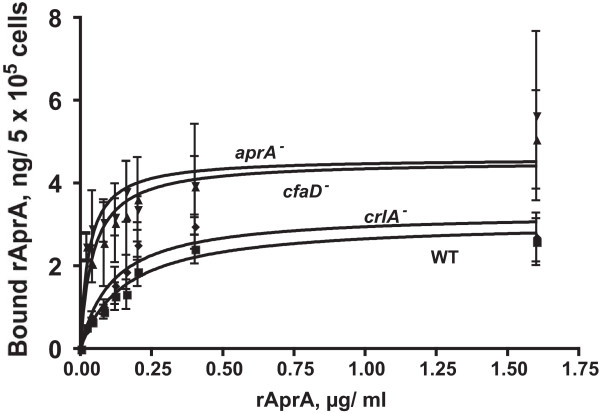
**Cells bind physiological concentrations of rAprA**. Cells of the indicated strains (WT is wild-type) were incubated with different concentrations of rAprA. After 10 minutes, cells were collected and the bound rAprA was measured as in Figure 5. The plot symbols are the same as in Figure 2. Values are mean ± SEM (n = 3). For the 60 kDa rAprA, a bound rAprA value of 1.0 ng/5 × 10^5 ^cells is equivalent to 2.0 × 10^4 ^molecules/cell. The lines are curve fits to a one-site binding model with no cooperative binding.

A key property of binding is that it is saturable. To examine whether the binding of rAprA to cells is saturable, cells were incubated with different concentrations of rAprA and the amount of bound rAprA was measured after 10 minutes. For wild-type and *crlA*^- ^cells, the binding of rAprA appeared to saturate above a free rAprA concentration of 0.4 μg/ml (Figure 6 and Table 3). There appeared to be a higher level of binding to *aprA*^- ^and *cfaD*^- ^cells, the binding appeared to roughly saturate, and there appeared to be a lower K_D _for binding to these two cell lines. The binding appeared to be specific, as competition with 10 μg/ml of BSA had no discernable effect on binding (data not shown). For all four cell lines, binding curves were fit using nonlinear regression with an equation for one-site binding. F-tests comparing these fits to fits with a two-site binding model, or fits to binding models with a variable Hill coefficient, indicated that for each of the four cell lines there did not appear to be two classes of binding sites or cooperative binding; the Hill coefficient for binding to wild-type cells was 1.0. Taken together, the data suggests that rAprA shows saturable binding to cells.

**Table 3:** The measured K_D _and Bmax for the binding of rAprA to vegetative cells.

**Cell type**	**K_D_, μg/ml**	**K_D_, nM**	**Bmax, ng/5 × 10^5 ^cells**	**Bmax, molecules/cell**
**WT**	0.16 ± 0.05	2.6 ± 0.8	3.1 ± 0.4	62,000 ± 8,000
***aprA*^-^**	0.03 ± 0.02	0.5 ± 0.3	4.6 ± 0.6	92,000 ± 11,000
***cfaD*^-^**	0.04 ± 0.03	0.7 ± 0.5	4.5 ± 0.7	91,000 ± 14,000
***crlA*^-^**	0.11 ± 0.04	1.9 ± 0.6	3.3 ± 0.3	66,000 ± 7,000

For each cell line, the K_D _in μg/ml and the Bmax in ng/5 × 10^5 ^cells was obtained from the curve fits in Figure 6. The K_D _in nM and Bmax in molecules/cell were calculated using a molecular mass of 60 kDa for rAprA. All values are mean ± SEM, n = 3. For both the the K_D_'s and the Bmax's, the difference between any two cell lines is not significant (p > 0.05, 1-way ANOVA, using either Tukey's or Bonferroni's test).

**Figure 7:**
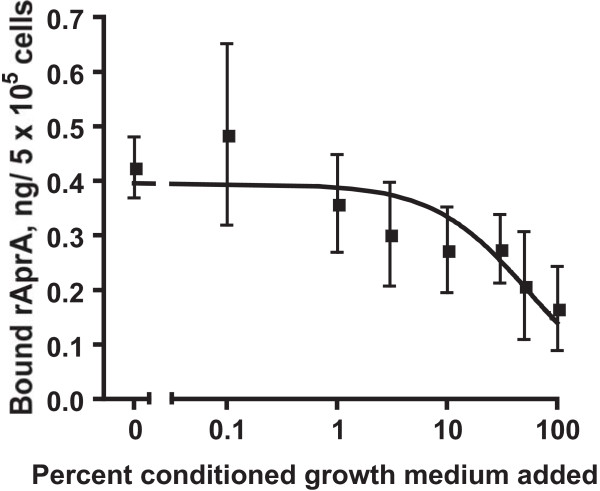
**Endogenous AprA competes with rAprA for binding to cells**. The binding of rAprA to *aprA*^- ^cells was measured in the presence of the indicated concentrations of wild-type conditioned growth medium from cells at 10^7 ^cells/ml, where the AprA concentration is 0.3 μg/ml. Values are mean ± SEM (n = 4). The line is a curve fit of a sigmoidal dose response curve.

To compare the cell surface binding of rAprA to that of native AprA, we measured the inhibition of rAprA binding to *aprA*^- ^cells by different concentrations of native AprA, using wild-type conditioned growth medium from cells at 1.2 × 10^7 ^cells/ml as a source of native AprA. As the concentration of wild-type conditioned growth medium in the binding assay increased, the amount of rAprA binding to *aprA*^- ^cells decreased (Figure 7). Using the equation of Cheng and Prusoff [34] and the observed AprA concentration of 0.3 μg/ml in wild type conditioned growth medium from cells at 1.2 × 10^7 ^cells/ml, we found that native AprA had an inhibition constant (K_i_) of 0.03 μg/ml, which is similar to the 0.03 μg/ml K_D _for the binding of rAprA to *aprA*^- ^cells. Taken together, the data suggest that the binding affinity of rAprA to *aprA*^- ^cells is roughly similar to that of native AprA.

## Discussion

*Dictyostelium *cells appear to regulate cell proliferation by secreting and sensing AprA and CfaD [29,30]. We found here that at 21°C, wild-type cells accumulate 260 molecules/cell/minute of extracellular AprA at low cell densities, and this decreases to 22 molecules/cell/minute as cells approach saturation density. Conversely, the CfaD accumulation rate appears to increase from less than 1 molecule/cell/minute at low density to 59 molecules/cell/minute near saturation density. These changes in accumulation rates as the cell density in a culture increases may be due to changes in nutrient or waste product levels, or changes in levels of signals such as AprA and CfaD. These accumulation rates are in the range of the 12 molecules/cell/minute for CMF [36], the 60 molecules/cell/minute for the accumulation of CF by wild-type cells [32], and the 250 molecules/cell/minute we observed for the accumulation of CF by *smlA*^- ^cells [20].

Using the AprA secretion rate of 49 molecules/cell/minute for cells harvested at 2 × 10^6 ^cells/ml, we can calculate that, if the binding assays had been done at 21°C, in our 10-minute binding assays wild-type cells would accumulate 4.9 × 10^-4 ^μg/ml of AprA. This is less than the lowest concentration of rAprA used for the binding curves (other than the buffer control) and well below the measured K_D_'s (Table 3). We saw that, at the density that we harvested cells for the binding assays, *crlA*^- ^and *cfaD*^- ^cells accumulated roughly 10 times more AprA than wild-type cells. This could be due to an increased AprA secretion rate or a decreased AprA degradation rate, or some combination of the two. Assuming a ten-times higher AprA secretion rate, *crlA*^- ^and *cfaD*^- ^cells would accumulate 4.9 × 10^-3 ^μg/ml of AprA in the 10-minute binding assays. This is still below the observed K_D_'s. At 4°C (the condition for the binding assays), the secretion rate will be even lower than at 21°C, so the amount of AprA secreted by cells during the binding assay should not have strongly interfered with the binding assay results.

Cells overexpressing Apra or CfaD still proliferate, albeit slowly, and recombinant CfaD slows but does not stop the proliferation of cells [29,30]. Like CfaD, rAprA slows but does not stop cell proliferation, even when combined with recombinant CfaD at concentrations three times higher than seen in the conditioned growth medium of stationary phase cells (Figures 2A and 2B). One can imagine that in the wild, a *Dictyostelium *cell might find itself in a small enclosed space where secreted factors might build up to very high concentrations, and having high concentrations of a chalone completely stop proliferation would be disadvantageous. The observed response of cells to AprA and CfaD (slowing but not stopping proliferation) thus might allow cells to increase their mass and protein content as they reach a high density, without incurring the risk of unnecessarily stopping proliferation under some conditions.

The response of wild-type and *aprA*^- ^cells is nonlinear: it takes approximately 0.01 μg/ml rAprA to decrease the cell density at 12 hours by 10%, approximately 0.1 μg/ml to decrease the density by an additional 10%, and more than 1 μg/ml to cause a further ~10% decrease (Figure 2A). rAprA appeared to have a higher activity (in units/μg) when added to *aprA*^- ^cells compared to its activity on wild-type cells (Figure 2A and Table 1). A qualitative explanation for this is that wild-type cells are accumulating extracellular AprA while *aprA*^- ^cells are not, so in the proliferation assay the wild-type cells are effectively starting at a higher extracellular AprA concentration compared to the *aprA*^- ^cells. Since our definition of a unit of AprA activity is the inverse of the amount of AprA needed to inhibit proliferation by 20% at 12 hours, and given the nonlinear response of cells to AprA, it would thus take more AprA to slow wild-type cells by an additional 20% compared to the amount of AprA needed to slow *aprA*^- ^cells by 20%. This would then predict that AprA would appear to have a lower activity on wild-type cells compared to its activity on *aprA*^- ^cells, which is what we observed.

Immunoprecipitated native extracellular AprA at 10 ng/ml significantly slowed the proliferation of wild-type and *aprA*^- ^cells [29], whereas higher concentrations of rAprA are needed to significantly slow proliferation. Native AprA has a higher molecular mass than recombinant AprA and thus appears to have some posttranslational modification, presumably glycosylation. This difference in posttranslational modifications may be the explanation for why native AprA appears to be more potent than recombinant AprA.

rAprA and rCfaD appear to potentiate each other's ability to inhibit proliferation (Figures 2A and 2B and Table 1). However, conditioned growth medium, which contains both AprA and CfaD, appears to have the same activity as rAprA, and thus has effectively less proliferation inhibiting activity than one would predict. This suggests that there may be some proliferation promoting activity in conditioned growth medium that counteracts the effects of AprA and CfaD. This proliferation promoting activity may well be due to a secreted growth factor activity (the factor has not been identified) that has been observed in *Dictyostelium *conditioned growth medium [37].

Wild-type cells show roughly steady-state binding after 10 minutes of incubation with rAprA, which is similar to the binding kinetics observed for countin [32] and conditioned medium factor (CMF) to *Dictyostelium *cells [16]. We found that recombinant AprA binds to wild-type cells with a K_D _of ~2.6 nM. This is stronger than the ~150 nM K_D _observed for folate binding to *Dictyostelium *cells [38], but quite similar to the 2.1 nM K_D _that we observed for CMF binding [16], and weaker than the 490 pM for CF50 binding [27], or 60 pM for countin binding [32]. Depending on the cell type, we observed ~6 – 9 × 10^4 ^AprA binding sites/cell. Although this is much higher than the ~50–60 countin or CF50 binding sites/cell [27,32], this is similar to the ~6 × 10^4 ^folate binding sites/cell [38] or ~4 × 10^4 ^CMF binding sites/cell [16]. Together, this suggests that the binding timecourse, K_D_, and number of binding sites/cell for AprA binding to cells are all within the range seen for the binding of other ligands to *Dictyostelium *cells. At 10^7 ^cells/ml, where the extracellular AprA concentration is 0.3 μg/ml, solving for the number of occupied cell-surface receptors using our observed K_D _and Bmax for wild-type cells, we see that there will be ~40,000 occupied receptors, or roughly 2/3 of the receptors will be occupied. This would then allow a strong activation of pathways downstream from the AprA receptor.

Although not statistically significant, it appears that *aprA*^- ^and *cfaD*^- ^cells have somewhat more AprA receptors than wild-type or *crlA*^- ^cells, and that the AprA receptors in *aprA*^- ^and *cfaD*^- ^cells have a lower K_D _(stronger binding) than the receptors in wild-type or *crlA*^- ^cells. A possible explanation for this is that there may be some degree of AprA-induced receptor desensitization and down regulation in wild-type and *crlA*^- ^cells, and that CfaD is necessary for this effect.

AprA and CfaD appear to be part of the same extracellular complex, and the presence of AprA is required for rCfaD to be able to slow proliferation [30], and conversely the presence of CfaD is needed for rAprA to slow proliferation (Figure 2A). However, rAprA shows roughly normal binding to cells in the absence of CfaD (Figures 4 and 5). This suggests that CfaD does not regulate AprA's proliferation-slowing activity by regulating its ability to bind to cells, but rather CfaD activates some pathway downstream of AprA binding that permits AprA signaling. This is strikingly similar to what we observed for countin and CF50, two protein components of the extracellular signal CF [27]. Countin and CF50 need each other for activity, but still bind to cells in the other's absence [27]. rAprA also needs the presence of the receptor-like protein CrlA to slow proliferation (Figure 2A), but surprisingly rAprA shows roughly normal binding to *crlA*^- ^cells (Figures 5 and 6). rCfaD slows the proliferation of *crlA*^- ^cells, although to a lesser extent than rCfaD slows wild-type or *cfaD*^- ^cells [30]. This suggests that CrlA is neither the AprA nor the CfaD receptor, but rather is part of a different pathway that for unknown reasons regulates the ability of AprA and CfaD to function as chalones to slow proliferation.

## Conclusion

Together, the data suggest that AprA functions as an autocrine proliferation-inhibiting factor by binding to cell surface receptors. Like CfaD, the concentration of AprA increases with cell density, and also like CfaD, AprA slows but does not completely stop proliferation. Although AprA requires CfaD for activity, and the two factors potentiate each other's activity, AprA does not require CfaD to bind to cells, suggesting the possibility that cells have an AprA receptor and a CfaD receptor, and activation of both receptors is required to slow proliferation. We previously found that *crlA*^- ^cells are sensitive to CfaD. Combined with the results presented here, this suggests that CrlA is not the AprA or CfaD receptor, and may be the receptor for an unknown third factor that is required for AprA and CfaD activity.

## Authors' contributions

JMC carried out the binding assays and drafted parts of the manuscript. DB prepared recombinant AprA, did the AprA quantitation assays, and drafted parts of the manuscript. JEP did proliferation inhibition assays. RHG conceived the study, participated in the design of the study, performed statistical analysis, and helped to draft the manuscript. All authors read and approved the final manuscript.
